# An information bottleneck-based optimal transport network for automated diagnosis of spinal diseases

**DOI:** 10.1038/s41746-026-02876-z

**Published:** 2026-07-10

**Authors:** Minghao Shao, Haocheng Xu, Linli Li, Yuan Dai, Fan Zhang, Hongli Wang, Jianyuan Jiang, Wei Zhu, Xiaosheng Ma, Feizhou Lyu

**Affiliations:** 1 Department of Orthopedics, Huashan Hospital, Fudan University, Shanghai, China; 2 Department of Orthopedics, Shanghai Fifth People’s Hospital, Fudan University, Shanghai, China

**Keywords:** Computational biology and bioinformatics, Diseases, Engineering, Health care, Mathematics and computing

## Abstract

Spinal diseases are common and widely impactful health issues in modern society. With the advancement of computer vision and medical image analysis, image-based automatic recognition and classification of spinal diseases have become research hotspots. However, existing methods often show limited performance in recognizing spinal diseases from complex or low-quality X-ray images. Their performance is easily affected by noise and exposure variations, leading to the extraction of pseudo-features unrelated to the disease. In addition, discrepancies among data sources and imaging conditions result in poor model generalization, making it difficult to adapt to cross-domain variations in clinical applications. To address these challenges, this study proposes an Information Bottleneck-based Optimal Transport Network (IBOTSpine) for automated diagnosis of spinal diseases. The IBOTSpine model introduces an information bottleneck-constrained feature extraction module that effectively captures disease-relevant structural information while suppressing irrelevant noise. Moreover, by incorporating an optimal transport mechanism, the model learns domain-invariant features, thereby reducing the distribution discrepancy between training and testing data and enhancing robustness and generalization across multi-source datasets. Specifically, the model employs a Swin Transformer as the backbone network and jointly optimizes the information bottleneck and optimal transport losses to achieve synergistic improvement in feature extraction, domain adaptation, and classification performance. Experimental results on real spinal X-ray dataset demonstrate that the proposed model outperforms existing methods in classification accuracy, generalization capability, and feature discriminability, validating its effectiveness and application potential in intelligent spinal image diagnosis.

## Introduction

Spinal diseases^1–3^ are common and widely impactful health problems in modern society, with complex pathogenesis that may be associated with multiple factors such as genetic predisposition, sedentary lifestyle, and long-term physical labor. Common spinal disorders include scoliosis^4,5^, intervertebral disc degeneration^6,7^, spondylolisthesis^8^, and osteoporosis^9^. Among them, idiopathic scoliosis is a prevalent structural abnormality, which can be diagnosed when the spinal curvature angle reaches or exceeds 10 degrees. If not detected and treated in time, patients may experience rapid disease progression during skeletal development, leading to pain, respiratory restriction, and physical deformities. Therefore, early screening and diagnosis of spinal diseases are of great clinical significance.

In current clinical practice, medical imaging examinations^10–12^ such as X-ray, CT, and MRI are the primary methods for evaluating spinal diseases. However, image interpretation heavily relies on the radiologist’s expertise and clinical experience, often requiring manual delineation of vertebral bodies or lesion regions and measurement of related parameters. This process is time-consuming and susceptible to subjective bias, which may lead to reduced diagnostic efficiency and inconsistent results. Moreover, with the increasing incidence of spinal diseases and limited medical resources, manual assessment alone can no longer meet the clinical demand for rapid and accurate diagnosis.

In recent years, with the advancement of computer vision and medical image analysis technologies, the automatic recognition and classification of spinal diseases using imaging data^13–18^ have become a research hotspot. By extracting structural features from X-ray and other imaging modalities, intelligent identification of various spinal abnormalities can be achieved, providing new solutions for large-scale screening and computer-aided diagnosis. Research in this field not only improves the efficiency of image interpretation but also offers technical support for early detection and precision treatment of spinal diseases.

At present, numerous studies have applied deep learning and other artificial intelligence methods to diagnose spinal diseases using X-ray or MRI images. For example, Haim et al.^19^ developed a diagnostic approach that integrates preoperative MRI images with postoperative pathological results to distinguish among various types of spinal disorders. Zhuo et al.^20^ proposed a deep learning-based predictive model that takes T2-weighted images as input and combines a multi-scale segmentation network with a feature extraction network to achieve automatic segmentation and classification of spinal cord lesions. This model can accurately differentiate between tumorous and demyelinating lesions as well as their subtypes. Degadwala et al.^21^ further introduced a model named DeepSpine, designed to classify multiple spinal diseases from X-ray images, including scoliosis, osteochondrosis, osteoporosis, spondylolisthesis, and compression fractures.

In addition, Kuang et al.^22^ proposed a hybrid supervised modeling strategy named SpineGEM for multi-pathology classification of lumbar degenerative diseases. The model integrates anatomical priors into a self-supervised pretraining framework, enabling high-accuracy classification with only a small amount of annotated data. Masood et al.^23^ developed an automated framework for lumbar spine disease assessment, capable of autonomously extracting spinal measurement parameters to assist clinical diagnosis. Using a ResNet-UNet architecture, the framework performs semantic segmentation of vertebral bodies and evaluates spondylolisthesis and lumbar lordosis based on angular deviation and curve area. Nguyen et al.^24^ constructed a large-scale annotated dataset containing over 10,000 spinal X-ray images, and established an image analysis framework named VinDr-SpineXR for the detection and localization of spinal abnormalities.

However, existing imaging-based diagnostic models for spinal diseases still suffer from several limitations. First, X-ray images are often affected by factors such as noise, exposure variation, and imaging angle. Traditional feature extraction modules struggle to capture disease-relevant structural information effectively, leading models to learn pseudo-features unrelated to the pathology. As a result, their recognition performance degrades significantly when dealing with complex or low-quality images. Second, the performance of these models varies considerably across different data sources, imaging devices, and patient populations, indicating that the learned features are domain-dependent and lack cross-domain generalization capability. When discrepancies exist between the training and testing sets in terms of image quality or lesion presentation, model robustness declines markedly, limiting its applicability in real clinical environments.

To address these challenges, this study proposes an Information Bottleneck-based Optimal Transport Network (referred to as IBOTSpine) for the automatic diagnosis of spinal diseases from X-ray images. The core idea of IBOTSpine is to employ an information bottleneck-enhanced feature extraction module^25–27^ to capture disease-related key information from complex spinal X-rays, thereby improving discriminative ability and diagnostic accuracy. Meanwhile, the model leverages an optimal transport mechanism^28–30^ to learn domain-invariant representations, effectively reducing the distribution gap between training and testing data and enhancing generalization performance across diverse data sources and imaging conditions.

Specifically, the proposed model simultaneously inputs spinal X-ray images from the training set (source domain) and the testing set (target domain) into a feature extraction module constrained by the information bottleneck, to obtain source-domain and target-domain features. This module, composed of a Swin Transformer backbone^31,32^ and an information bottleneck loss, preserves discriminative information while suppressing irrelevant noise. Subsequently, the source-domain features are fed into a classifier to predict spinal disease categories, with classification performance optimized through cross-entropy loss. At the same time, both source and target features are passed into an optimal transport module, which computes the distributional distance and constrains the network via an optimal transport loss, ensuring better alignment of the two domains in latent space. Finally, the model is trained under a joint optimization objective that combines cross-entropy loss, optimal transport loss, and information bottleneck loss, achieving synergistic improvements in feature extraction, domain adaptation, and diagnostic performance.

Experimental results on real real-world spinal X-ray dataset demonstrate that the IBOTSpine model outperforms existing methods in terms of classification accuracy, generalization capability, and feature discriminability, validating the effectiveness and practical value of the proposed framework.

The main contributions of this study can be summarized as follows. We propose a spinal disease diagnostic framework that integrates information bottleneck and optimal transport mechanisms to achieve synergistic optimization of feature extraction and domain adaptation. An information bottleneck-constrained Swin Transformer module is designed to effectively suppress noisy features and highlight pathology-related regions. In addition, an optimal transport mechanism is introduced to align cross-domain feature distributions, significantly enhancing the model’s robustness and generalization capability across different data sources. Finally, experimental results on real spinal X-ray dataset demonstrate the superior diagnostic accuracy and clinical applicability of the proposed model.

## Results

In this study, we propose an Information Bottleneck-based Optimal Transport Network (hereafter referred to as IBOTSpine) for the automatic diagnosis of spinal diseases from X-ray images. The core idea of the model is to employ a feature extraction module enhanced by information bottleneck constraints to capture disease-relevant key information from complex spinal X-rays, thereby improving the model’s discriminative capability and diagnostic accuracy. Meanwhile, the IBOTSpine model leverages an optimal transport mechanism to learn domain-invariant features, effectively reducing the distribution gap between the training and testing sets and enhancing the model’s generalization performance across different data sources and imaging conditions.

**Fig. 1:**
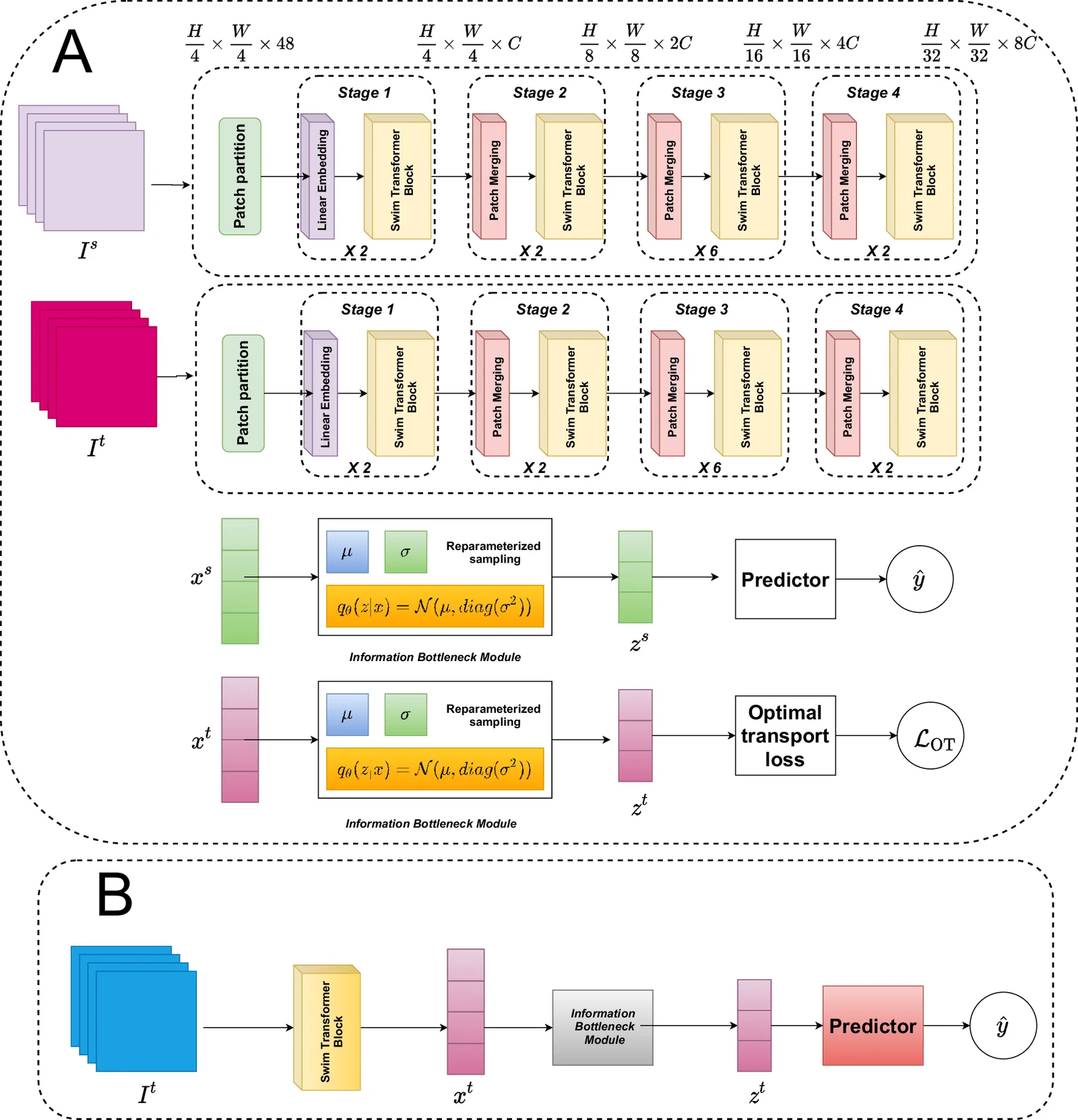
The training and inference pipeline of the proposed IBOTSpine model for spinal disease diagnosis. **A** Training stage; **B** Inference stage.

Figure 1 illustrates the training and inference processes of the IBOTSpine model. Specifically, the model simultaneously inputs spinal X-ray images from the training set (source domain) and testing set (target domain) into the information bottleneck-constrained feature extraction module to obtain source and target features. At the same time, these features are fed into the optimal transport module, which computes the distributional distance and constrains the network through optimal transport loss, ensuring greater consistency between the two domains in the latent space.

### Model comparison with baselines

To evaluate the performance of the proposed model, the IBOTSpine network was compared with several representative spinal image classification models, including ResNet, DenseNet, EfficientNet, ViT, ConvNeXt, CoAtNet, EfficientFormer, and Xception. All models were trained under identical data preprocessing, training protocols, and optimization settings to ensure a fair comparison. The evaluation metrics included Precision, Accuracy, F1-score, Recall, AUC, and AUPR.

Experiments were conducted under two different data partition strategies to comprehensively assess model robustness: a 7:3 split and a 5:5 split on the Vertebrae X-ray Dataset.

**Fig. 2:**
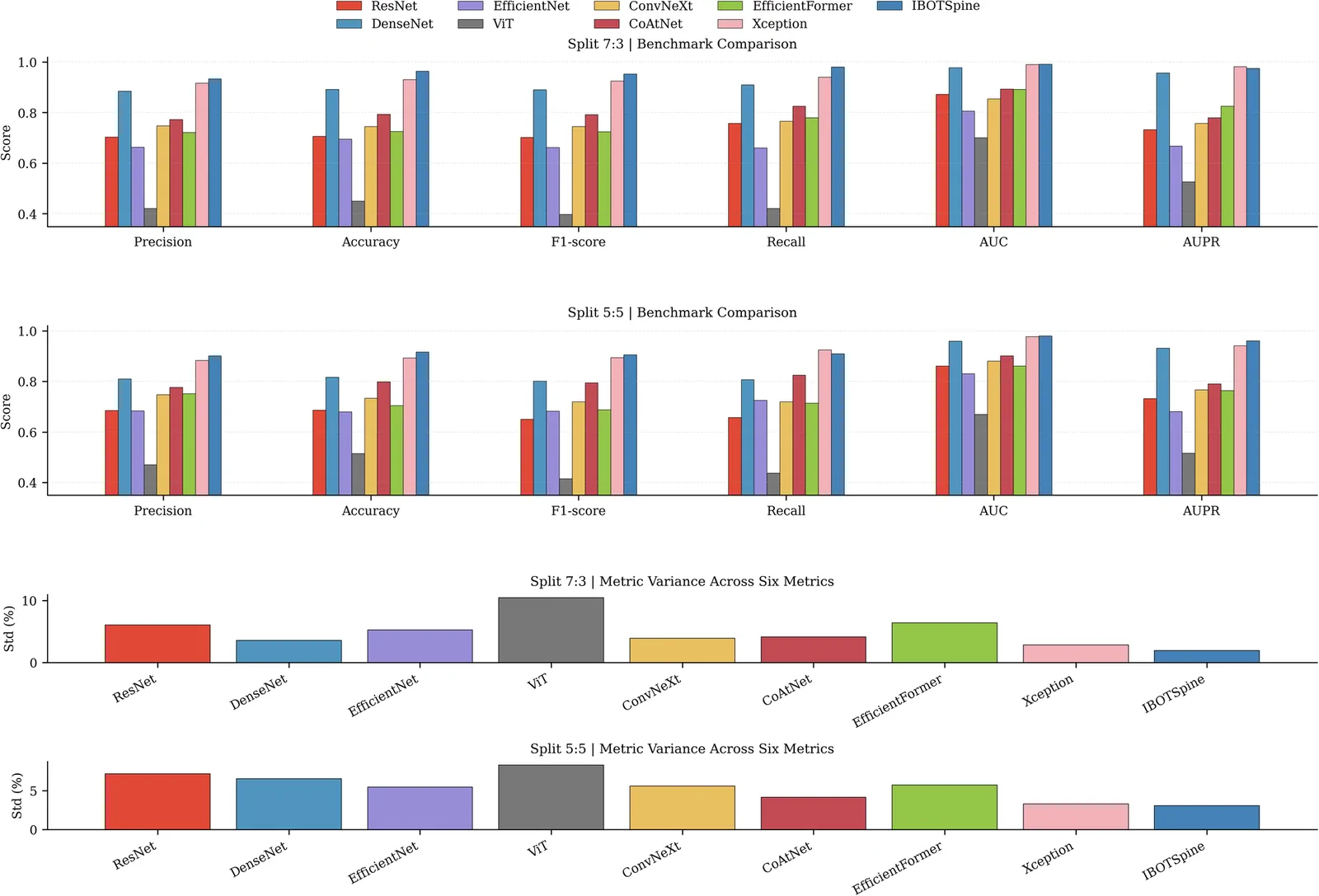
Performance comparison of the proposed IBOTSpine model with baseline on spinal X-ray dataset. Results are reported for two data splits (7:3 and 5:5) across six evaluation metrics, including Precision, Accuracy, F1-score, Recall, AUC, and AUPR, along with the corresponding metric variance.

As shown in Table 1 and Fig. 2, the proposed IBOTSpine model consistently achieved the best performance across all evaluation metrics under both settings. On the Vertebrae X-ray Dataset (7:3 split), IBOTSpine achieved a Precision of 0.933, Accuracy of 0.964, F1-score of 0.953, Recall of 0.980, AUC of 0.991, and AUPR of 0.975, outperforming all competing methods. Compared with the strongest baseline (Xception), IBOTSpine improved Accuracy from 0.931 to 0.964 and F1-score from 0.925 to 0.953, demonstrating enhanced classification capability.

**Table 1:** Performance comparison of IBOTSpine and baseline methods under two split settings

Vertebrae X-ray Dataset (7:3)						
Model	Precision	Accuracy	F1-score	Recall	AUC	AUPR
ResNet	0.703	0.706	0.703	0.758	0.873	0.732
DenseNet	0.886	0.892	0.891	0.910	0.977	0.957
EfficientNet	0.664	0.696	0.662	0.660	0.806	0.668
ViT	0.421	0.451	0.398	0.422	0.701	0.527
ConvNeXt	0.747	0.745	0.745	0.766	0.855	0.757
CoAtNet	0.773	0.794	0.792	0.825	0.893	0.780
EfficientFormer	0.721	0.725	0.725	0.780	0.893	0.825
Xception	0.917	0.931	0.925	0.941	0.990	0.982
**IBOTSpine (ours)**	**0.933**	**0.964**	**0.953**	**0.980**	**0.991**	**0.975**

Vertebrae X-ray Dataset (5:5)						
Model	Precision	Accuracy	F1-score	Recall	AUC	AUPR
ResNet	0.686	0.686	0.651	0.658	0.861	0.733
DenseNet	0.811	0.817	0.802	0.808	0.960	0.932
EfficientNet	0.684	0.680	0.683	0.726	0.831	0.681
ViT	0.470	0.515	0.415	0.438	0.670	0.516
ConvNeXt	0.747	0.734	0.719	0.720	0.881	0.768
CoAtNet	0.777	0.799	0.794	0.826	0.901	0.791
EfficientFormer	0.752	0.704	0.688	0.714	0.861	0.764
Xception	0.883	0.893	0.894	0.924	0.977	0.941
**IBOTSpine (ours)**	**0.901**	**0.917**	**0.905**	**0.910**	**0.981**	**0.961**

Bold values: our own model's results.

On the Vertebrae X-ray Dataset (5:5 split), IBOTSpine also demonstrated consistent superiority, achieving a Precision of 0.901, Accuracy of 0.917, F1-score of 0.905, Recall of 0.910, AUC of 0.981, and AUPR of 0.961. Compared with Xception, IBOTSpine further improved Accuracy from 0.893 to 0.917 and AUC from 0.977 to 0.981, indicating improved robustness and generalization under a more challenging data partition.

Overall, the IBOTSpine model achieved the best performance across both datasets, demonstrating superior accuracy and stability compared to all baseline methods. This improvement can be attributed to the introduction of information bottleneck constraints in the feature extraction stage, which effectively emphasize disease-relevant key information while suppressing irrelevant noise, and to the optimal transport mechanism, which enables the learning of domain-invariant features, thereby reducing distributional discrepancies across imaging conditions and devices and significantly enhancing the model’s generalization ability and diagnostic reliability.

### Ablation study

In this section, we conduct an ablation study to investigate the effect of the OT loss on the performance of the IBOTSpine model. The OT loss weight is varied within the range of [0,0.005,0.01,0.05,0.2].

**Fig. 3:**
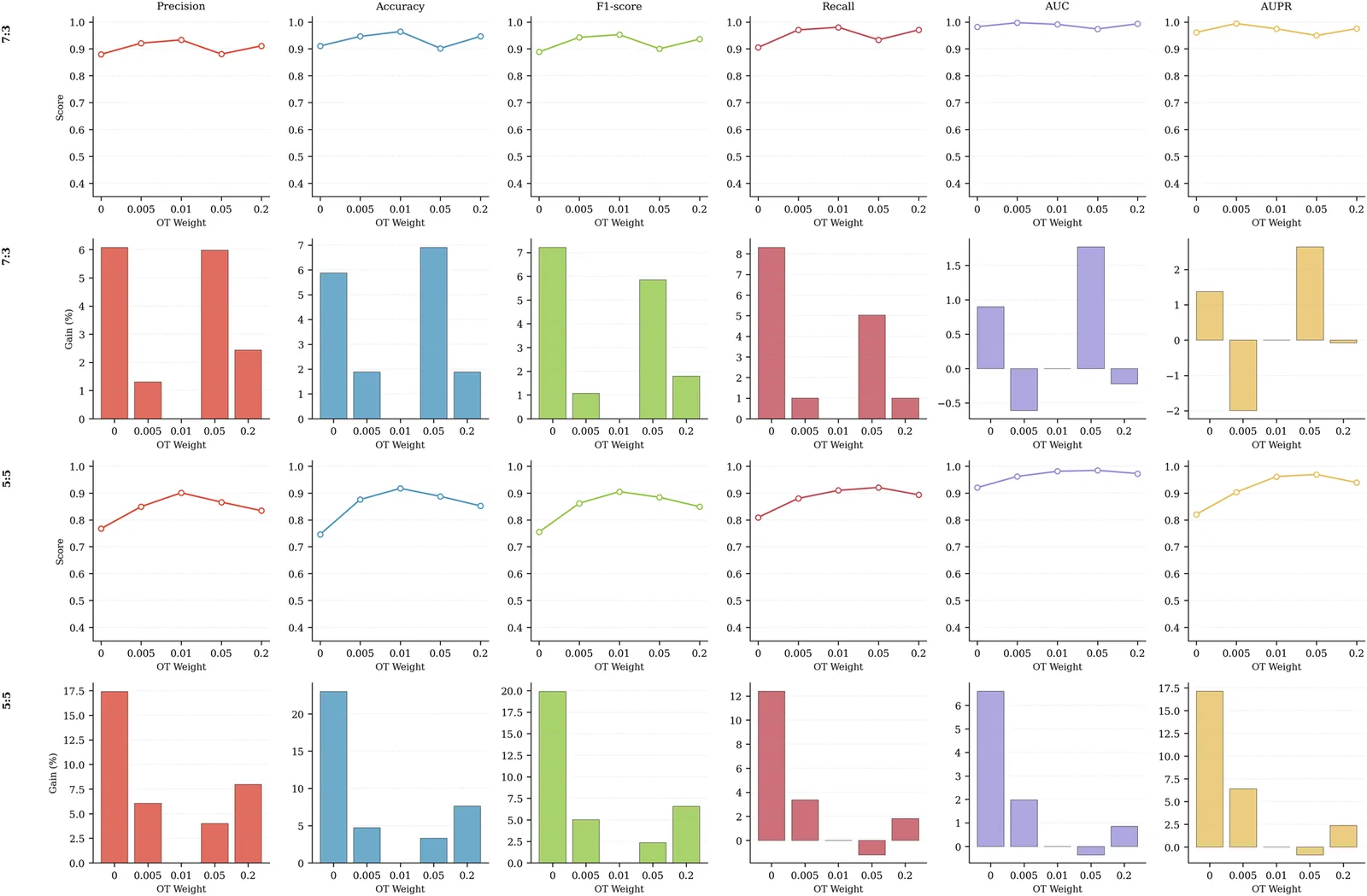
Ablation experiments analyzing the effects of the optimal transport loss. The performance is evaluated under different OT weights across two data splits (7:3 and 5:5) using six metrics (Precision, Accuracy, F1-score, Recall, AUC, and AUPR), along with the corresponding performance gain.

The experimental results are presented in Table 2 and Fig. 3. Overall, introducing the OT loss consistently improves model performance compared to the baseline setting without OT (weight = 0) under both data split strategies, demonstrating its effectiveness in enhancing feature alignment and classification performance.

**Table 2:** Ablation study on the effects of different weights for the optimal transport loss under two split settings

Vertebrae X-ray Dataset (7:3)						
OT Weight	Precision	Accuracy	F1-score	Recall	AUC	AUPR
0 (without OT)	0.880	0.911	0.889	0.905	0.982	0.961
0.005	0.921	0.946	0.943	0.971	0.997	0.994
**0.01**	**0.933**	**0.964**	**0.953**	**0.980**	**0.991**	**0.975**
0.05	0.881	0.902	0.900	0.933	0.974	0.950
0.2	0.911	0.946	0.936	0.971	0.993	0.975

Vertebrae X-ray Dataset (5:5)						
OT Weight	Precision	Accuracy	F1-score	Recall	AUC	AUPR
0 (without OT)	0.767	0.746	0.755	0.809	0.920	0.820
0.005	0.849	0.876	0.862	0.880	0.962	0.903
**0.01**	**0.901**	**0.917**	**0.905**	**0.910**	**0.981**	**0.961**
0.05	0.866	0.888	0.884	0.921	0.984	0.969
0.2	0.834	0.852	0.849	0.893	0.972	0.939

Bold values indicate the best overall performances achieved by our model under the corresponding OT weight condition.

On the Vertebrae X-ray dataset (7:3 split), the model achieves the best overall performance when the OT weight is set to 0.01, with a Precision of 0.933, Accuracy of 0.964, F1-score of 0.953, AUC of 0.991, and AUPR of 0.975. In contrast, without OT (weight = 0), the performance is significantly lower, with Precision of 0.880, Accuracy of 0.911, F1-score of 0.889, AUC of 0.982, and AUPR of 0.961. As the OT weight increases beyond the optimal value, the performance becomes less stable. For example, when the weight is 0.2, although the AUC remains relatively high (0.993), the overall classification metrics, including Precision (0.911) and F1-score (0.936), decline compared to the optimal setting.

A similar trend is observed under the 5:5 split setting. When the OT weight is 0.01, the model achieves the best performance, with Precision of 0.901, Accuracy of 0.917, F1-score of 0.905, AUC of 0.981, and AUPR of 0.961. Without OT, the performance drops substantially, with Precision of 0.767, Accuracy of 0.746, F1-score of 0.755, AUC of 0.920, and AUPR of 0.820. Increasing the OT weight to 0.2 leads to a decline in performance (Precision: 0.834, Accuracy: 0.852, F1-score: 0.849, AUC: 0.972), indicating that excessive weighting negatively impacts the model.

These results reveal a clear trade-off in the use of OT loss. A moderate OT weight (0.01) effectively improves feature alignment and enhances generalization across different data splits. However, overly large weights may introduce excessive distribution constraints, which can suppress task-relevant discriminative features and ultimately degrade classification performance.

### Visualization analysis

**Fig. 4:**
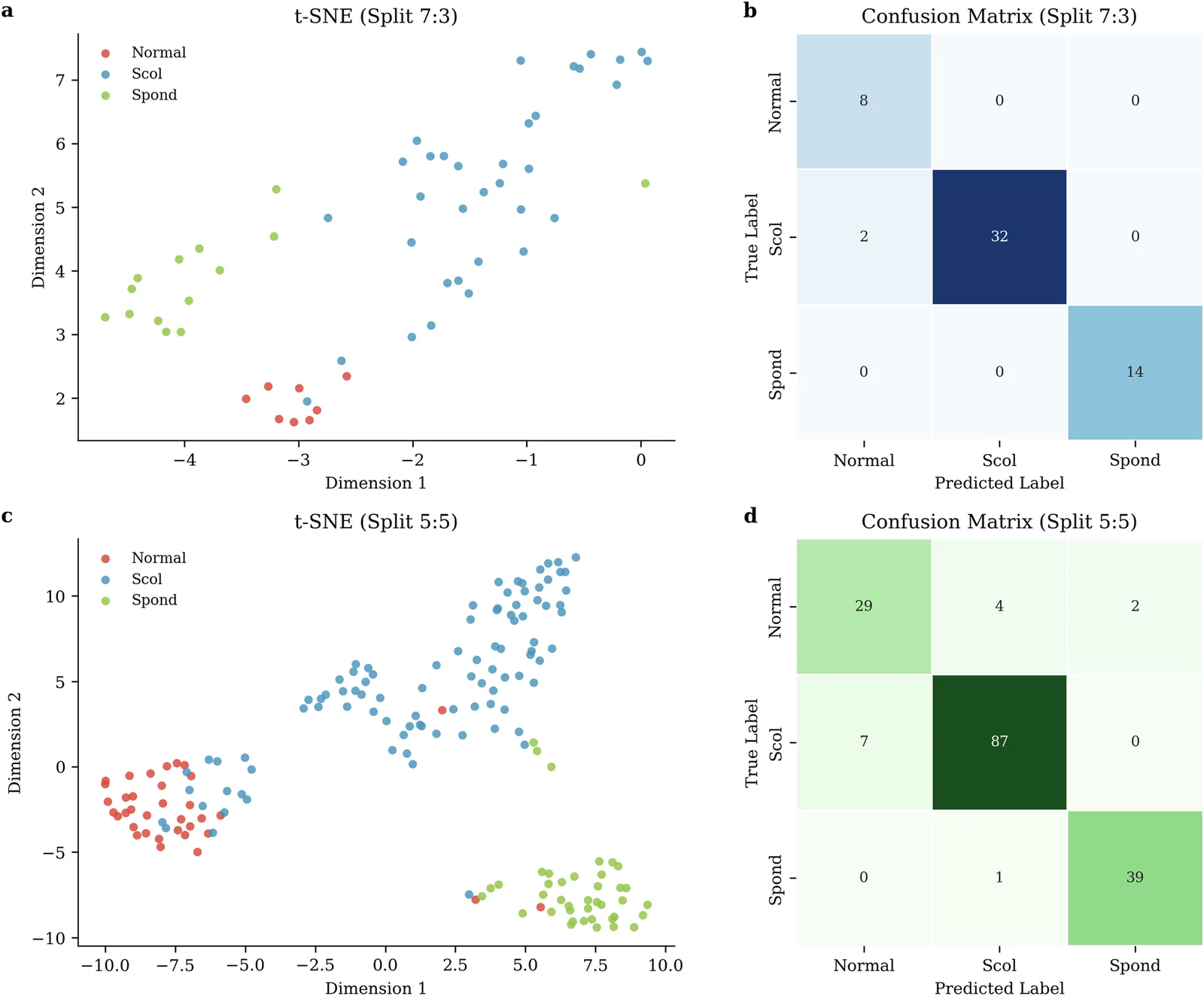
t-SNE visualizations and confusion matrices illustrating the discriminative power and interpretability of IBOTSpine. **a**, **c** t-SNE embeddings for data splits 7:3 and 5:5, respectively; **b**, **d** corresponding confusion matrices illustrating classification performance.

**Fig. 5:**
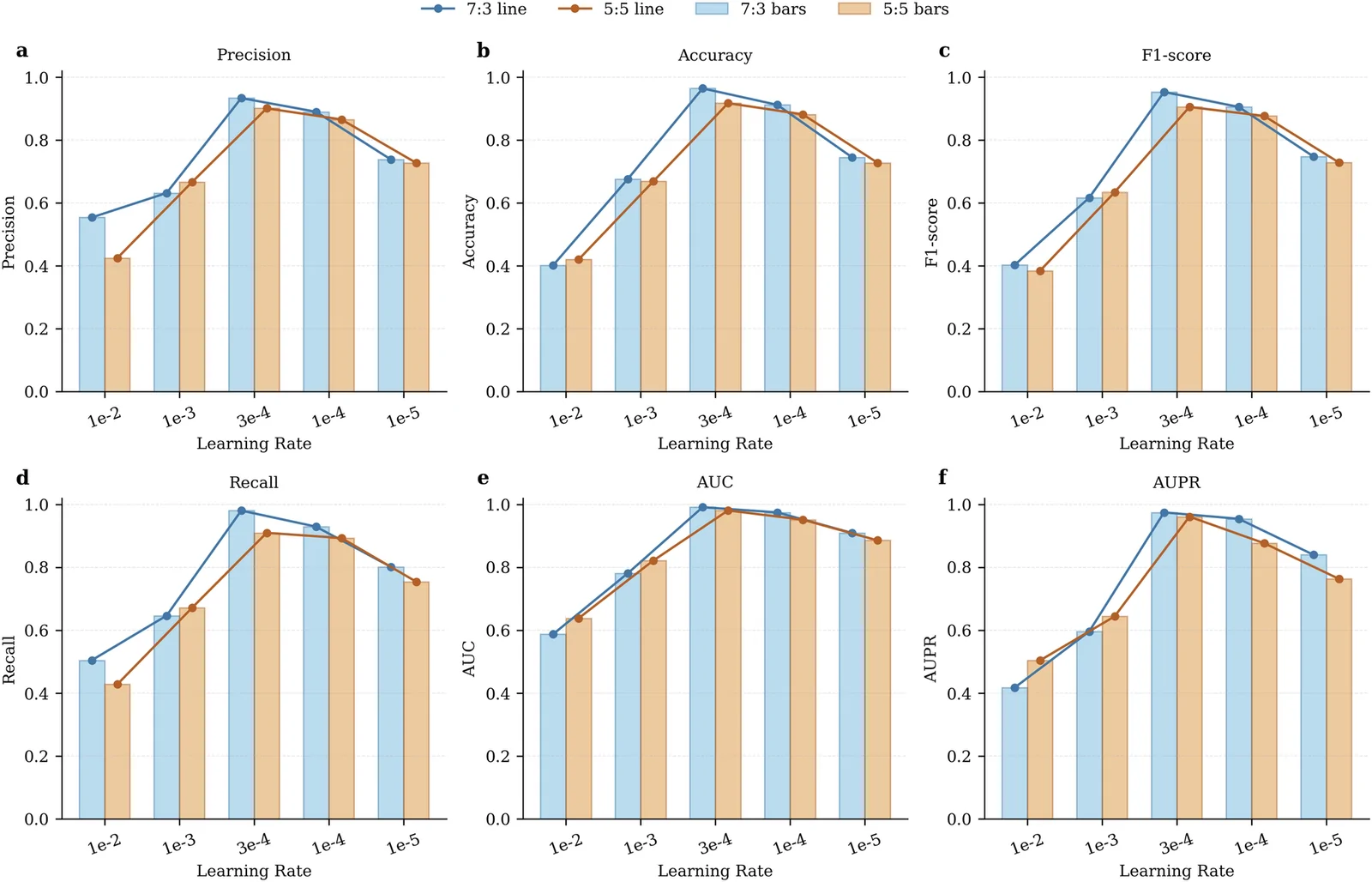
Effect of learning rate on model performance. **a**–**f** Precision, Accuracy, F1-score, Recall, AUC, and AUPR under different learning rates for two data splits (7:3 and 5:5).

In this section, we conduct a visual analysis of the feature representations learned by the IBOTSpine model for spinal disease classification. Figure 4 presents t-SNE visualizations and confusion matrices illustrating the discriminative power and interpretability of IBOTSpine. As illustrated in Fig. 5a and Fig. 5a, the t-SNE projections under both the 7:3 and 5:5 split settings exhibit clear clustering patterns for different disease categories. Specifically, samples from the Normal, Scoliosis (Scol), and Spondylolisthesis (Spond) classes form compact and well-separated clusters with minimal overlap, indicating that the learned feature embeddings possess strong discriminative power.

Moreover, the cluster structure remains stable across different data splits, suggesting that the model captures robust and consistent feature representations. Notably, even under the more challenging 5:5 split, where training data is reduced, the inter-class separation is still well preserved, further demonstrating the generalization capability of IBOTSpine.

### Hyperparameter sensitivity analysis

In this section, we analyze the sensitivity of the IBOTSpine model to the learning rate hyperparameter. The learning rate is varied over a wide range from 1 × 10^−5^ to 1 × 10^−2^ to investigate its impact on model performance.

As shown in Table 3 and Fig. 5, the performance of IBOTSpine is highly sensitive to the choice of learning rate under both data split settings. The model achieves the best overall performance when the learning rate is set to 3 × 10^−4^. Specifically, under the 7:3 split, IBOTSpine attains a Precision of 0.933, Accuracy of 0.964, F1-score of 0.953, Recall of 0.980, AUC of 0.991, and AUPR of 0.975. Similarly, under the 5:5 split, the model achieves a Precision of 0.901, Accuracy of 0.917, F1-score of 0.905, Recall of 0.910, AUC of 0.981, and AUPR of 0.961, indicating consistent optimal performance across different data partitions.

**Table 3:** Sensitivity analysis of IBOTSpine to different learning rates under two split settings

Vertebrae X-ray Dataset (7:3)						
Learning Rate	Precision	Accuracy	F1-score	Recall	AUC	AUPR
1e-2	0.554	0.402	0.402	0.505	0.588	0.417
1e-3	0.632	0.676	0.617	0.646	0.781	0.596
**3e-4**	**0.933**	**0.964**	**0.953**	**0.980**	**0.991**	**0.975**
1e-4	0.890	0.912	0.905	0.929	0.975	0.954
1e-5	0.738	0.745	0.748	0.802	0.909	0.840

Vertebrae X-ray Dataset (5:5)						
Learning Rate	Precision	Accuracy	F1-score	Recall	AUC	AUPR
1e-2	0.425	0.420	0.384	0.428	0.638	0.504
1e-3	0.666	0.669	0.634	0.671	0.822	0.644
**3e-4**	**0.901**	**0.917**	**0.905**	**0.910**	**0.981**	**0.961**
1e-4	0.865	0.882	0.877	0.893	0.951	0.877
1e-5	0.727	0.728	0.729	0.754	0.886	0.763

Bold values indicate the best overall performances achieved by our model under the corresponding OT weight condition.

When the learning rate is too large (e.g., 1 × 10^−2^), the model fails to converge effectively, resulting in significantly degraded performance (Accuracy drops to 0.402 and 0.420 under the 7:3 and 5:5 splits, respectively). As the learning rate decreases to 1 × 10^−3^, the performance improves but remains suboptimal. When the learning rate is further reduced to 1 × 10^−4^, the model achieves relatively strong performance; however, it still falls short of the optimal setting.

On the other hand, when the learning rate becomes too small (e.g., 1 × 10^−5^), the model exhibits degraded performance again, likely due to slow convergence and insufficient optimization, as reflected by reduced Accuracy (0.745 and 0.728 under the two splits).

Overall, these results demonstrate that IBOTSpine achieves the best trade-off between convergence stability and generalization performance at a moderate learning rate (3 × 10^−4^). Excessively large learning rates lead to unstable training, while overly small learning rates hinder effective optimization and result in suboptimal performance.

## Discussion

The proposed IBOTSpine model is designed to address two long-standing challenges in automated spinal image diagnosis. The first challenge lies in the difficulty of feature extraction caused by noise, exposure variations, and structural overlap in X-ray images. The second challenge concerns domain shifts arising from differences in imaging devices and population sources. By incorporating an information bottleneck constraint and an optimal transport mechanism into the Swin Transformer backbone, the model demonstrates significant advantages in both feature extraction and cross-domain generalization.

From the experimental results, the IBOTSpine model consistently outperforms existing mainstream networks on real spinal imaging dataset. Compared with convolutional architectures such as ResNet, DenseNet, and EfficientNet, IBOTSpine makes more effective use of multi-scale contextual information. In contrast to architectures like Vision Transformer and ConvNeXt, the introduction of the information bottleneck constraint suppresses the model’s reliance on non-pathological features, making the extracted representations more focused on lesion-relevant regions. Moreover, the optimal transport module adaptively aligns latent feature distributions across different domains, thereby significantly improving the model’s generalization capability under heterogeneous data sources.

The ablation studies further illustrate the effects of the mechanisms. When the weight of the optimal transport loss is too small, the model fails to adequately align cross-domain features, leading to reduced generalization. Conversely, an excessively large weight may weaken the learning of discriminative features. The best performance achieved around a loss weight of 0.01 indicates that distribution alignment is critical for optimal model performance.

The learning rate sensitivity analysis reveals that the IBOTSpine model achieves stable convergence and optimal performance when the learning rate is set to 3Ã10^−4^. A smaller learning rate results in slow convergence, while a larger one induces oscillations and overfitting. This observation aligns with prior findings regarding the training stability of Transformer-based architectures in medical imaging tasks, further validating the robustness of the proposed framework.

In summary, this study demonstrates the effective integration of information bottleneck and optimal transport mechanisms for spinal disease image diagnosis. The proposed IBOTSpine model not only improves discriminative performance but also enhances stability under varying imaging conditions. Future work will consider incorporating multimodal data (e.g., CT and MRI) and pathology-based auxiliary labels to further improve the model’s clinical applicability and decision reliability.

## Methods

In this study, we propose an Information Bottleneck-based Optimal Transport Network (IBOTSpine) for the automated diagnosis of spinal diseases from X-ray images. As illustrated in Fig. 1, the core idea of the model is to extract disease-relevant key information from complex spinal X-rays through a feature extraction module enhanced by an information bottleneck constraint, thereby improving the model’s discriminative ability and diagnostic accuracy. Meanwhile, the IBOTSpine model employs an optimal transport mechanism to learn domain-invariant features, effectively reducing the distribution gap between the training and testing sets and enhancing the model’s generalization capability across different data sources and imaging conditions.

### IBOTSpine model

Figure 1 a presents the training process of the IBOTSpine model, while Fig. 1b illustrates its inference process.

Specifically, during the training phase, the input data of the IBOTSpine model consist of X-ray samples from the **training set***I*^*s*^ (source domain) and the **testing set***I*^*t*^ (target domain). As shown in Equations (1–2), the IBOTSpine model first feeds *I*^*s*^ and *I*^*t*^ into the **feature learning module** based on the **Swin Transformer**, thereby obtaining their corresponding feature representations *x*^*s*^ and *x*^*t*^.

1 $${x}^{s}={f}_{{\rm{Swin}}}({I}^{s}),$$

2 $${x}^{t}={f}_{{\rm{Swin}}}({I}^{t}),$$

Here, *f*_Swin_( ⋅ ) denotes the feature extraction function constructed by the Swin Transformer, *I*^*s*^ and *I*^*t*^ represent the spinal X-ray image inputs from the source domain (training set) and the target domain (testing set), respectively, while *x*^*s*^ and *x*^*t*^ correspond to their feature representations in the high-dimensional latent space.

Subsequently, the IBOTSpine model feeds *x*^*s*^ and *x*^*t*^ into the **information bottleneck module** to obtain the compressed features *z*^*s*^ and *z*^*t*^. As shown in Equations (3–4), for the features *x*^*s*^ and *x*^*t*^, two sets of linear transformations are applied to derive the mean *μ* and the log-variance *σ*^2^, respectively.

3 $${{\boldsymbol{\mu }}}^{s}={W}_{\mu }{x}^{s}+{b}_{\mu },\quad \log {{\boldsymbol{\sigma }}}^{s2}={W}_{\sigma }{x}^{s}+{b}_{\sigma },$$

4 $${{\boldsymbol{\mu }}}^{t}={W}_{\mu }{x}^{t}+{b}_{\mu },\quad \log {{\boldsymbol{\sigma }}}^{t2}={W}_{\sigma }{x}^{t}+{b}_{\sigma },$$

Here, *W*_*μ*_ and *W*_*σ*_ denote the learnable linear transformation matrices, while *b*_*μ*_ and *b*_*σ*_ are the corresponding bias terms; ***μ*** and ***σ*** represent the mean and standard deviation of the feature distributions, respectively; and the symbol ⊙ indicates element-wise multiplication. Through this parameterization, the model can represent the uncertainty of input features in the form of a Gaussian distribution.

To maintain network differentiability, as shown in Equations (5–6), the model employs the *reparameterization trick* to sample the latent variables *z*^*s*^ and *z*^*t*^ from the learned distributions. This step not only performs information compression but also enhances feature robustness through stochastic sampling while preserving gradient backpropagation, thereby providing more stable representations for subsequent classification and domain alignment tasks.

5 $${z}^{s}={{\boldsymbol{\mu }}}^{s}+{{\boldsymbol{\sigma }}}^{s}\odot {{\boldsymbol{\epsilon }}}^{s},\quad {{\boldsymbol{\epsilon }}}^{s} \sim {\mathcal{N}}(0,I),$$

6 $${z}^{t}={{\boldsymbol{\mu }}}^{t}+{{\boldsymbol{\sigma }}}^{t}\odot {{\boldsymbol{\epsilon }}}^{t},\quad {{\boldsymbol{\epsilon }}}^{t} \sim {\mathcal{N}}(0,I),$$

Equation (7) presents the information bottleneck loss, which enables the model to suppress irrelevant noise while preserving discriminative information.

7 $${{\mathcal{L}}}_{{\rm{IB}}}={D}_{{\rm{KL}}}\,\left({q}_{\theta }(z| x)\,\parallel \,p(z)\right)=\frac{1}{2}\mathop{\sum }\limits_{j=1}^{d}\left({\mu }_{j}^{2}+{\sigma }_{j}^{2}-\log {\sigma }_{j}^{2}-1\right),$$

Here, *D*_KL_( ⋅ ∣ ⋅ ) denotes the Kullback-Leibler (KL) divergence, *q*_*θ*_(*z*∣*x*) represents the posterior distribution learned by the model, $p(z)={\mathcal{N}}(0,I)$ is the prior distribution, and *d* is the dimensionality of the latent variable.

The information bottleneck loss constrains the latent variable distribution to be close to a standard normal distribution, thereby suppressing disease-irrelevant noise features while preserving discriminative information. In this way, the model focuses on key features related to spinal structures and lesion regions, enhancing both the compactness and discriminability of the feature representations.

Next, as shown in Equation (8), the IBOTSpine model feeds *z*^*s*^ into a fully connected layer to predict the spinal disease category $\hat{y}$. Equation (9) presents the cross-entropy loss.

8 $$\hat{y}={\rm{Softmax}}({W}_{c}{z}^{s}+{b}_{c}),$$

9 $${{\mathcal{L}}}_{{\rm{CE}}}=-\mathop{\sum }\limits_{i=1}^{C}{y}_{i}\log ({\hat{y}}_{i}),$$

Moreover, as shown in Equation (10), the IBOTSpine model feeds *z*^*s*^ and *z*^*t*^ into the optimal transport loss, where the difference between their vector means is used as an approximation. By inputting the source and target domain features into the optimal transport module, the model computes the distribution distance and constrains the network through the optimal transport loss, thereby making the features from both domains more consistent in the latent space.

10 $${{\mathcal{L}}}_{{\rm{OT}}}={\left\Vert {\mathbb{E}}\,\left[{z}^{s}\right]-{\mathbb{E}}\,\left[{z}^{t}\right]\right\Vert }_{2}^{2},$$

Here, ${\mathbb{E}}[{z}^{s}]$ and ${\mathbb{E}}[{z}^{t}]$ denote the mean vectors of the latent variables in the source and target domains, respectively, and $| \cdot {| }_{2}^{2}$ represents the squared L2 norm. This formulation approximates the optimal transport distance using the Euclidean distance between the mean feature representations of the two domains, effectively measuring the distributional discrepancy in the latent space.

Finally, as shown in Equation (11), the model is trained with a joint optimization objective that combines the cross-entropy loss, the optimal transport loss, and the information bottleneck loss, thereby achieving synergistic improvements in feature extraction, domain adaptation, and diagnostic performance.

11 $${{\mathcal{L}}}_{{\rm{total}}}={{\mathcal{L}}}_{{\rm{CE}}}+\beta \,{{\mathcal{L}}}_{{\rm{IB}}}+\lambda \,{{\mathcal{L}}}_{{\rm{OT}}},$$

By jointly minimizing the above loss functions, the model is able to suppress noisy features and align cross-domain feature distributions while maintaining classification accuracy. This achieves a well-balanced trade-off among feature extraction, domain adaptation, and diagnostic performance, thereby significantly enhancing the robustness and generalization capability of spinal disease recognition.

During the inference phase, the IBOTSpine model directly inputs an X-ray image into the corresponding modules to obtain the final prediction.

### Dataset

In this study, we utilized a publicly available spinal X-ray image dataset^33^ for spinal disease classification tasks. The dataset, referred to as the Spinal Disease Classification Dataset, was collected from the affiliated hospital of Jordan University of Science and Technology. It contains spinal X-ray images from 338 subjects (240 females and 98 males), covering three categories: normal, spondylolisthesis, and scoliosis.

To comprehensively evaluate model performance under different data availability conditions, we designed two experimental settings using different training-testing split ratios. Specifically, the dataset was divided into training and testing sets using a 7:3 ratio and a 5:5 ratio, respectively. The 7:3 split reflects a standard supervised learning setup with relatively sufficient training data, while the 5:5 split simulates a more challenging scenario with limited training samples. These two configurations enable a systematic assessment of the model’s robustness and generalization capability.

## Data Availability

The data used in this study are publicly available and were not generated by the authors. The dataset can be accessed at DOI:10.17632/xkt857dsxk.1.

## Code availability

The code used in this study is available from the corresponding author upon reasonable request.
